# Radiation Therapy for Locally Recurrent Breast Cancer

**DOI:** 10.1155/2012/571946

**Published:** 2012-10-03

**Authors:** Joshua Siglin, Colin E. Champ, Yelena Vakhnenko, Pramila R. Anne, Nicole L. Simone

**Affiliations:** Department of Radiation Oncology, Kimmel Cancer Center, Jefferson Medical College, Thomas Jefferson University, Philadelphia, PA 19107, USA

## Abstract

Approximately one-third of all breast cancer patients experience local recurrence of their tumor after initial treatment. As initial treatment often employs the use of radiation therapy (RT), the standard of care for local breast cancer recurrence after initial breast conserving therapy has traditionally been surgical intervention with mastectomy. However, recent attempts to preserve the intact breast after recurrence with local excision have revealed a potential need for RT in addition to repeat breast conserving surgery as rates of local failure with resection alone remain high. Additionally, local recurrence following initial mastectomy and chest wall RT can be treated with reirradiation to increase local control. Repeating RT, however, in a previously irradiated area, is a complex treatment strategy, as the clinician must carefully balance maximizing treatment effectiveness while minimizing treatment-related toxicity. As a result, physicians have been hesitant to treat recurrent disease with repeat RT with limited data. Results from the current literature are promising and current clinical trials are underway to explore reirradiation modalities which will provide additional information on treatment-related toxicity and outcomes. This paper will review the current literature on repeat radiation therapy for locally recurrent breast cancer.

## 1. Introduction

Of the nearly 300,000 cases of invasive and in situ breast cancers diagnosed in 2010 alone, 20–40% of these patients will experience a local recurrence (LR) and approximately half of those will have isolated recurrences [1]. Optimal local control is noted to be important for patient outcomes. Local recurrences still occur at rates reported in large, randomized trials between 3–20% rate for patients receiving breast conserving therapy (BCT) and a 2–12% for patients who have undergone mastectomy [2–4]. Accordingly, treatment of locally recurrent breast cancer remains important in the care of breast cancer patients. Patient and tumor characteristics, as well as initial treatment, have a significant impact on the treatment options offered for recurrent disease. Given the heterogeneous nature of local breast cancer recurrence, several treatment options are available and in this paper we will further outline the role of radiation therapy (RT) which is one such treatment modality.

## 2. Recurrence in Patients Treated Initially with BCT

Mastectomy remains the standard of care for patients with in-breast tumor recurrence (IBTR) after initial treatment with BCT. Mastectomy in the setting of recurrent disease results in a second local recurrence rate of 2–31% [5]. In patients with an in-breast tumor recurrence (IBTR), five-year overall survival rates after mastectomy range from 52 to 84% in various series [6–10]. While mastectomy remains the current standard of care for IBTR, a growing body of literature describes repeat BCT in the setting of locally recurrent disease. 

### 2.1. Repeat Breast Conserving Therapy without Reirradiation

More women are choosing to preserve their breast in the setting of recurrence with repeat BCT. Initial experiences with BCT for recurrence focused on partial mastectomy alone without repeat irradiation. Kurtz et al. reported on 52 patients who had received wide local excision for isolated LR. Patients with mobile tumors less than 2 cm in diameter and no signs of rapid progression were carefully selected for breast preservation. In this group, the five-year local control and cause-specific survival were 79% which are comparable to results noted with mastectomy. Interestingly, it was noted that local control was doubled when negative margins were achieved with local excision on second surgery. Therefore, it seems that the potential exists to avoid a mastectomy in the setting of recurrent disease if adequate margins are obtained [11].

This was explored further by Salvadori et al. in a large series reporting on patients receiving a mastectomy or a local resection for isolated LR. With a median followup of 6 years, the risk of a second local recurrence was significantly increased in the local resection group at 19% versus 4% for total mastectomy [6]. These results were echoed by Abner et al. and Voogd et al., showing a LR rate of 31% at 3.5 years and 38% at 4.5 years, respectively, with a local resection alone (Table 1) [12–14].

While these rates of recurrence are significantly greater than those from total mastectomy, they are comparable to the 35% risk of recurrence found in upfront breast conserving surgery without the addition of RT. This has led investigation into the feasibility of repeat irradiation to the IBTR recurrence after resection to minimize the risk of local recurrence. To date, repeat radiation has been described using various treatment techniques such as external beam radiation therapy (EBRT), intraoperative radiation therapy (IORT), and brachytherapy.

## 3. Second Breast Conserving Therapy with Repeat Radiation Therapy

Overall, the published data on reirradiation have shown promising results. In terms of local control, reported rates range from 77–100%, with the limited available prospective data revealing a very favorable 7–11% rate of second LR. Furthermore, acceptable rates of toxicity and cosmesis have been obtained in all reported series, regardless of variations in RT technique for repeat partial breast irradiation. The methodologies previously utilized in the literature will be reviewed, along with the associated outcomes data.

### 3.1. External Beam Radiation Therapy

As early as the mid-1980s, EBRT was employed to reirradiate patients with breast cancer recurrence. Deutsch has reported on approximately 40 women with IBTR following partial mastectomy and radiation therapy. Initial RT consisted of 50 Gy to the whole breast in 2 Gy fractions with or without tumor bed boost. At the time of recurrence, a median of 63 months from initial treatment, partial breast reirradiation (PBrI) to a dose of 50 Gy in 2 Gy daily fractions was delivered to the entire operative quadrant using electrons of appropriate energy. Deutsch showed that among 39 patients at a median followup of 52 months, 77% of patients had an intact breast free of tumor. Treatment was well tolerated but alteration in skin pigmentation was reported as well as 25% of evaluated patients having fair to poor cosmesis [15].

A heterogeneous group of 29 patients receiving reirradiation with 3D-conformal RT were reviewed by Würschmidt et al., of which 4 received second breast conserving therapy. Cosmetic results were good to excellent with no Grade 3 or higher toxicity in this small group of patients [16].

**Figure 1:**
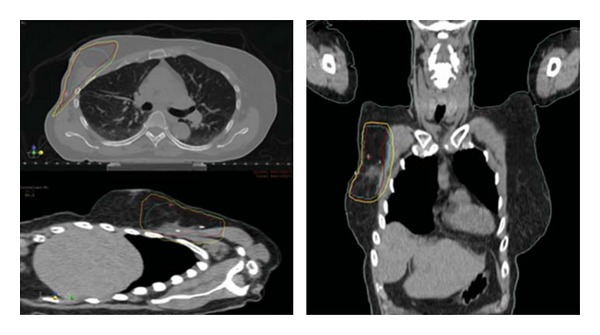
Partial breast radiation therapy plan utilizing external beam radiation therapy with photons. Blue: planning target volume- evaluation (PTVeval), red: 95% isodose line, yellow: 90% isodose line.

The Radiation Therapy Oncology Group (RTOG) is evaluating a similar treatment approach with external beam partial breast radiation therapy in its current phase II trial. The radiation volume will consist of the surgical margin plus CTV and PTV expansions to generate a 2.5 cm margin excluding the skin surface. This will result in a significantly smaller treatment volume (Figure 1) than the initial RT volume, which usually encompasses the entire breast. Twice daily fractions of 1.5 Gy will be administered to a total dose of 45 Gy.

### 3.2. Intraoperative Radiation Therapy

Single institution data on IORT utilized the Intrabeam device, with a median applicator size of 4.0 cm (Carl Zeiss, Oberkochen, Germany), which delivered a median dose of 20 Gy to the applicator surface. In this study, postprocedure margins were noted to range from 1 to 10 mm and after more than 2 years of followup no local recurrences were noted. IORT was also well tolerated with no Grade 3 or higher toxicity and a majority of excellent or good cosmetic outcomes [17].

### 3.3. Interstitial Brachytherapy

The use of interstitial brachytherapy as a method of delivering focal radiation in the setting of locally recurrent breast cancer has been well documented. A French retrospective study of almost 70 patients who underwent repeat lumpectomy followed by interstitial brachytherapy revealed promising 5-year OS and freedom from second local recurrence (FFLR2). Despite the dissimilar treatment modality, Hannoun-Levi et al. showed results similar to those of Deutsch. A 77% rate of freedom from second local recurrence was noted in 70 patients. Interestingly, in this group of patients treated with interstitial brachytherapy, multivariate analysis revealed the subset of patients treated with 5 or more wires had a markedly improved FFLR2 of nearly 95% at 5 years. This treatment course delivered a median of 7.5 years from initial diagnosis and was also well tolerated with minimal acute toxicity. A 10% rate of late Grade 3 toxicity was noted, most commonly fibrosis [18]. While only in abstract form, a much larger retrospective series of 217 patients receiving second conservative treatment with lumpectomy and interstitial brachytherapy was reported on at the World Congress of Brachytherapy in 2012. The 5-year local recurrence rate was a promising 5.6% with actuarial OS at 5 years reported as 88.7%. Furthermore, cosmesis was good to excellent in 85% of patients with an 11% rate of Grade 3 and 4 toxicity noted [19].

While the aforementioned retrospective data provides insight on the safety and efficacy of PBrI, limited prospective data exist for reirradiation in the setting of locally recurrent breast cancer. Three prospective trials have reported encouraging local control rates, with all studies employing brachytherapy techniques to deliver a second course of radiation therapy. A phase II trial from Austria enrolled 39 patients with IBTR who refused mastectomy and opted for a second breast conserving treatment. Similarly, Guix et al. conducted a study of a second breast conserving treatment in 36 women with limited-size, low-risk local recurrence occurring at a greater than 12 month interval who had refused mastectomy. Patients were excluded from both studies if LR was within 12 months of initial treatment. There was a median interval between treatments of nearly 11 years in the study by Kauer-Dorner et al., but only 3.2 years in the Guix et al. study.

Radiation therapy techniques also differed in these two trials. The Austrian group utilized multicatheter interstitial pulsed dose rate (PDR) brachytherapy following an R0 repeat resection. Mean physical, total dose of 50.1 Gy in fractions of 0.6 to 1 Gy was prescribed. Of note, 10 patients had received chemotherapy and 24 received hormonal therapy. The Spanish group employed an HDR technique to deliver 30 Gy in 12 fractions over a period of 5 days. Adjuvant chemotherapy was given to 13 patients, while 8 patients received hormonal therapy [20, 21].

Additionally, a smaller phase I/II study utilized low dose rate brachytherapy, initially to a total dose of 30 Gy in 6 patients. The dose was escalated to 45 Gy for the subsequent 9 patients as toxicity was minimal. Consistent with the majority of data, an admixture of systemic therapy was reported with a portion of patients receiving chemotherapy or hormonal therapy [22].

In this phase I/II trial, Chadha et al. reported an 89% local control rate, but patient numbers were low and median followup was only 36 months. However, similar 5-year actuarial local control of 93% was seen in the larger Austrian study with a median followup of 57 months. In this study, 5-year OS and DFS were 87% and 77%, respectively. Ten-year actuarial data were reported in the Spanish study, showing an OS of 96.7%, DFS of 64.4%, and local control of 89.4%.

Guix et al. reported low treatment-related toxicity, with no Grade 3 or 4 events reported. Only 4% of patients in this study had unsatisfactory cosmetic outcomes. Kauer-Dorner et al. did note Grade 3 breast tissue fibrosis in 1 woman and 3 women had Grade 3 pain. Poor or unacceptable cosmesis was documented in 6 patients by independent observers; however, only 2 patients self-reported this level of cosmetic outcome. Thus equivalent local tumor control to mastectomy was obtained by second BCT with good cosmesis and moderate morbidity. Overall, the addition of PBrI to repeat breast conserving surgery appears to be both efficacious and well tolerated (Table 2). However, when considering the use of a second breast conserving therapy it is important to remember that the study populations were highly selected patients with small local recurrences occurring more than one year from initial treatment [20–22].

### 3.4. Future Directions

Further study of repeat BCT employing surgery and reirradiation with partial breast is currently underway in a phase II trial, RTOG 1014. This study will evaluate the adverse events associated with the use of PBrI in patients with recurrent breast cancer following a margin-negative repeat lumpectomy. As in the Austrian study, recurrence within 12 months is an exclusion criterion. External beam radiation therapy is being administered to a total dose of 45 Gy using a 1.5 Gy twice daily fractionation. The study will secondarily evaluate endpoints of local control, freedom from mastectomy, cosmesis and OS.

## 4. Recurrence in Patients Who Received Initial Mastectomy

Guidelines for the treatment of local recurrence in patients treated initially with mastectomy are slightly more complex than for those patients who received upfront BCT. The standard of care is currently surgical resection, if technically feasible. Similar to patients who had prior BCT, systemic therapy is to be considered. In patients who have not previously received RT, the NCCN Guidelines recommend chest wall and nodal irradiation. There is a large body of literature demonstrating the use of radiation therapy for chest wall recurrence, including data on chest wall reirradiation. However, caution is warranted in repeat chest wall RT as aggregate doses of 100 Gy or higher can pose significant risk of toxicity such as skin ulceration, brachial plexopathy, osteonecrosis, rib fracture, and cardiomyopathy.

### 4.1. Chest Wall Radiation Therapy without Prior RT

In the context of patients who had not had prior radiation, the addition of chest wall radiation therapy at the time of recurrence has well-documented utility for improvement of local control [23, 24]. Halverson et al. reported the Washington University experience with chest wall radiation therapy for local-regional recurrence following mastectomy [25]. Patients treated to a large RT field encompassing the entire chest wall, FFR, were 75% and 63% at 5 and 10 years, compared with 36% and 18% in patients treated to a smaller field. In this study, field size proved to be an important factor for freedom from recurrence (FFR) and it was recommended that the entire chest wall is treated. A radiation dose of 60 Gy seemed to adequately control tumors less than 3 cm in this study, while only half of larger tumors were controlled by doses of up to 70 Gy.

Another single institution retrospective review [26] published by Hsi et al. treated patients at the Hospital of the University of Pennsylvania with a chest wall recurrence following mastectomy using radiation to a median chest wall dose of 50 Gy along with 10 Gy scar boost. At a median followup of 8.4 years, the ten-year actuarial local control was 86%, overall survival 72%, and cause specific survival 77% [27]. It was found that patients who had greater than 2-year disease-free interval after mastectomy, isolated chest wall recurrence, a tumor less than 3 cm, or a completely resected tumor, seemed to respond most favorable to radiation.

### 4.2. Repeat Chest Wall Radiation Therapy

While the addition of chest wall RT for patients with local recurrence in patients without previous RT is fairly straightforward, repeat irradiation is more controversial due to the high cumulative doses that would be achieved and potential toxicity. Local control with repeat surgery alone remains unacceptable with 5-year local control rates of only 33% [28]. Subsequently, the use of repeat chest wall RT has been explored for a second curative intent treatment despite the concerns over possible toxicity.

A multi-institutional study by Wahl et al. reviewed the feasibility of chest wall reirradiation. Eighty-one patients received an initial median dose of 60 Gy and after local recurrence underwent a second course of RT to the chest wall. Median dose for the second treatment course was 48 Gy. Two-thirds of patients were free of local disease at 12 months, with the local DFS rate significantly higher in patients without gross disease. Overall, the complete response rate was 57% [29]. Three Grade 3 late toxicities occurred, with patients experiencing fibrosis, infection, and lymphedema. The lone Grade 4 event, dermatitis, occurred in a patient who had a cumulative RT dose of ≥120 Gy.

A German retrospective study from the University of Tübingen was recently published by Muller et al. reviewing data on the role of repeat surgery and chest wall reirradiation for curative intent. Forty-two women had received an initial median dose of 54 Gy and after local recurrences received either surgery followed by chest wall reirradiation or chest wall reirradiation alone. The second course of radiation therapy was conventionally fractionated to a median dose of 60 Gy. The median interval between courses of RT was slightly longer than that reported by Wahl et al., 53 months versus 38 months. At a median followup of 41 months, the estimated 5-year local control was 62% and 5-year OS was 59%. Only 2 cases of acute Grade 3 skin toxicity were noted. Eight cases of late Grade 3 skin toxicity were reported and no acute or late Grade 4 toxicity occurred [30].

These studies indicate that chest wall reirradiation can provide an improved prognosis for women with local recurrence and can be administered with acceptable acute and late toxicities following initial treatment of breast cancer with surgery and postoperative radiation therapy.

## 5. Conclusion

Despite a growing body of literature, the role of reirradiation for locally recurrent breast cancer remains ill-defined. Repeat breast conserving therapy has shown promise in retrospective reviews and small prospective studies. The role of PBrI will hopefully be further elucidated by more prospective studies, including the ongoing RTOG trial. In patients who have previously received chest wall RT following mastectomy, treatment of local recurrence with reirradiation has been shown to be feasible. Given the heterogeneity in the recurrent breast cancer patient population, reirradiation remains an individualized treatment decision.

## Figures and Tables

**Table 1:** Outcomes of patients treated with breast conserving surgery alone following in-breast local recurrence.

Study	Number of patients	Median followup (months)	Local recurrence (%)	5-year overall survival (%)
Kurtz et al. [11]	55	51	32	NR
Abner et al. [12]	16	39	31	81
Salvadori et al. [6]	57	73	19	85
Voogd et al. [13]	16	52	38	NR
Ishitobi et al. [14]	78	40	21.2	NR

NR: not reported.

**Table 2:** Outcomes of patients treated with repeat breast conserving therapy following in-breast local recurrence.

Study	Number of patients	Median followup (months)	RT technique	Repeat RT dose (Gy)	≥ Grade 3 toxicity (%)	Local control (%)	Overall survival (%)
Deutsch [15]	39	51.5	EBRT	50*	NR	76.9	77.9^‡^
Hannoun-Levi et al. [18]	69	50.2	Brachytherapy	30 or 45–50	10.2	77.4	91.8^‡^
Kraus-Tiefenbacher et al. [17]	15	26	IORT	20*	0	100	93.3^¥^
Würschmidt et al. [16]	4	NR	NR	NR	0	NR	NR
Chadha et al. [22]	15	36	Brachytherapy	30 or 45	0	89	100^*¥*^
Guix et al. [21]	36	89	Brachytherapy	30	0	89.4**	96.7**
Kauer-Dorner et al. [20]	39	57	Brachytherapy	50.1^†^	16.7	93	87^‡^

NR: not reported, Gy: Gray, RT: radiation therapy, EBRT: external beam radiation therapy, IORT: intraoperative radiation therapy.

*Median, ^†^mean, ^‡^at 5 years, **10-year actuarial, ^¥^at median followup.
